# Experiences of UK and Irish family carers of people with profound and multiple intellectual disabilities during the COVID-19 pandemic

**DOI:** 10.1186/s12889-023-17432-7

**Published:** 2023-12-11

**Authors:** Mark Linden, R Leonard, T Forbes, M Brown, L Marsh, S Todd, N Hughes, M Truesdale

**Affiliations:** 1 School of Nursing and Midwifery, The Queen’s University of Belfast, 97 Lisburn Road, Belfast, Northern Ireland BT9 7BL UK; 2 School of Healthcare Sciences, University of South Wales, Cardiff, UK; 3 ESRC Centre for Care, Department of Sociological Studies, University of Sheffield, Sheffield, UK; 4 School of Health and Wellbeing, University of Glasgow, Glasgow, UK

**Keywords:** Intellectual disability, Family carers, COVID-19, Qualitative, Focus groups

## Abstract

**Background:**

People with profound and multiple intellectual disabilities (PMID) have high and intensive support needs that ordinarily place significant strain on family carers. This was further heightened by the removal of many external supports during the COVID-19 pandemic. We sought to explore the experiences of family carers of people with PMID during the COVID-19 pandemic and understand what the longer-term impact might be on their lives.

**Methods:**

Focus group interviews (n = 32) were conducted with family carers (n = 126) from the four countries of the UK and the Republic of Ireland. Participants were asked questions relating to their experiences of the COVID-19 pandemic, coping strategies, and challenges faced. All focus groups were conducted using the online platform, Zoom. These were audio recorded, transcribed verbatim and analysed employing inductive thematic analysis.

**Findings:**

Three main themes were generated from the data including (1.0) COVID-19 as a double-edged sword (2.0), The struggle for support (3.0), Constant nature of caring. These included 11 subthemes. (1.1) ‘COVID-19 as a catalyst for change’, (1.2) ‘Challenges during COVID-19: dealing with change’, (1.3) ‘Challenges during COVID-19: fear of COVID-19’, (1.4); ‘The online environment: the new normal’ (2.1) ‘Invisibility of male carers’, (2.2) ‘Carers supporting carers’, (2.3) ‘The only service you get is lip service: non-existent services’, (2.4); ‘Knowing your rights’ (3.1) ‘Emotional response to the caring role: Feeling devalued’, (3.2) ‘Emotional response to the caring role: Desperation of caring’, (3.3) ‘Multiple demands of the caring role.’

**Conclusions:**

The COVID-19 pandemic presented immense challenges to family carers of people with PMID but also provided some opportunities. Families had already struggled to receive many of the supports and services to which they were entitled to only to have these removed at the onset of the pandemic. The experiences of male carers have been largely absent from the literature with this research showing they want to be included in decision making and require tailored support services. Service providers should see the end of the COVID-19 pandemic as providing opportunity to re-examine current provision and design services with family carers. As the direct threat from COVID-19 diminishes and the experiences of those who lived through this period come to the fore, there is a need to re-examine current models and provision of support to family carers to better meet their needs.

## Introduction

Profound and multiple intellectual disabilities (PMID) refers to a condition where individuals have extremely delayed intellectual and social functioning and limited verbal communication, require someone to interpret their needs and may have additional medical, physical or sensory impairments [1]. People experiencing PMID have high support needs and will most likely require 24-hour-a-day support with all aspects of their lives including personal care, washing and dressing, eating, medical care as well as needing meaningful activity throughout the day. Family carers of people with PMID therefore provide continuous care across the lifespan for their family member.

During the COVID-19 pandemic many family carers were forced to juggle new caregiving demands, and some had to do so whilst also facing the challenges of working from home [2]. For some family carers who were frontline, or essential workers, managing these changes to their established roles resulted in increased strain [3]. This was particularly noted in cases where family members were medically vulnerable and their primary carer may have increased the risk of exposure to COVID-19 due to their occupation [4]. Some carers were forced to live separately from their families, which placed increased burden on those families [5].

Family carers of those with disabilities faced numerous challenges during the COVID-19 pandemic. Research has shown that the removal of some services and supports during the COVID-19 pandemic and lockdown restrictions led to increased stress, anxiety and depression [6–8]. An online, international survey conducted with 1,912 family carers of people with intellectual and developmental disabilities showed high levels of stress and depression which were predicted by the mood of the person they cared for [7]. However, some family carers described how the COVID-19 pandemic had a positive impact on their family life by providing dedicated time to learn new skills and strengthen family bonds [9, 10]. For example, qualitative research conducted with five mothers of children with intellectual disability (ID) in the Netherlands during the first lock down period showed how mothers were forced to make the changing situation work but also how strong bonds with their partners helped them cope [10].

Our parallel study sought to capture the particular lived experiences of family carers of people with PMID during the COVID-19 pandemic, and to compare these experiences across the UK and the Republic of Ireland (RoI). The varied and contrary experiences of family carers of those with disabilities were thought likely to be particularly pronounced in the context of PMID, and therefore worthy of specific attention. Comparing experiences in countries with varied support structures and responses to the pandemic provided a richer understanding of the impact of the pandemic and of supports received, and provided opportunity to re-examine current provision and design services with family carers of PMID. Indeed, the findings presented here have subsequently underpinned the development of a support programme for family carers of people with PMID (www.carers-ID.com).

## Methods

### Design

This manuscript describes the second phase of data collection in a larger study which explored the experiences of family carers of people with PMID. Phase one, explored family carer experiences through the lens of non-government organisations (NGOs) who worked closely to support them before, during and after the COVID-19 pandemic [11]. The findings included here (phase 2) are those of the family carers themselves. Utilising a qualitative focus group methodology we sought to understand how family carers across the UK and The RoI had experienced the COVID-19 pandemic.

### Participants

This research employed focus group interviews with 126 family carers across the four countries of the UK and the RoI. Participants were recruited through NGOs who had existing relationships with family carers. Eight-nine participants identified as female while nineteen identified as male. Whilst 91% of participants were of working age (18–65 years), only 16% (n = 17) were in full time employment. The majority of family carers (74%, n = 80) provided more than 10 h of care. Participants reported on 128 ethnicities indicating that many held multiple ethnicities. However, the majority (78.1%, n = 100) identified as white. We talked to family carers across the UK and Ireland; specifically, Scotland (n = 19), Wales (n = 13), England (n = 32), Northern Ireland (n = 30), and the Republic of Ireland (n = 13). Participants were provided with a £20 gift certificate as a payment in thanks for their time. Characteristics of 108 study participants who provided their demographic details can be found in Table 1.

**Table 1:** Demographics of included participants (n = 108/126)

*Variable name*	*N(%)*
*Gender*	
Female	89 (82%)
Male	19 (18%)
***Age ranges***	
Under 18	1 (1%)
18–24	2 (2%)
25–34	10 (9%)
35–44	29 (27%)
45–54	30 (28%)
55–64	26 (24%)
65–74	8 (7%)
75–84	2 (2%)
***Region***	
England	32 (30%)
Scotland	19 (17%)
Wales	13 (12%)
Northern Ireland	30 (28%)
Republic of Ireland Missing data	13 (12%) 1 (1%)
***Ethnicity***	
White	100 (78.1%)
White and Black African	11 (8.6%)
Any other mixed/multiple ethnic background	1 (0.8%)
Indian	2 (1.6%)
Pakistani	3 (2.3%)
Bangladeshi	1 (0.8%)
African	5 (3.9%)
Any other Black/African/Caribbean background	3 (2.3%)
Other	2 (1.6%)
***Average hours caregiving per day***	
More than 15	60 (56%)
10–15	20 (18%)
5–10	14 (13%)
1–5 Missing data	11 (10%) 3 (3%)
***In receipt of carers benefits***	
Yes	50 (46%)
No Missing data	55 (51%) 3 (3%)
***In paid employment***	
Yes – Full time	17 (16%)
Yes – Part time	34 (31%)
No Missing data	54 (50%) 3 (3%)

### Data collection

Thirty-two focus groups with family carers were conducted between March and December 2022. Family carers were recruited through NGOs who had worked closely with them during the COVID-19 pandemic. These organisations acted as gatekeepers for the project and emailed letters of invitation to family carers to inform them of the project, followed by a more detailed information sheet approximately one week later. Those who expressed an interest in participating in the study shared their details with the gatekeeper who arranged a suitable time for the focus group to take place. Due to the distributed geographical location of participants, focus groups were held via Zoom. Our NGOs reported that family carers were most familiar with using Zoom during the COVID-19 pandemic and were proficient with this platform. The online approach also afforded greater flexibility of timings to coincide with carers’ responsibilities, required no travel time and brought together carers who had not previously met. Further, we felt that speaking from the comfort of their own homes would be beneficial in eliciting a relaxed and open account of carers’ experiences. Focus group interviews were recorded and converted to audio files for the purposes of transcription. A transcription service was employed to produce accurate transcripts. Basic demographic details were gathered by means of an online questionnaire. The focus group topic guide included questions on family carers’ experiences of the pandemic, challenges, coping strategies and supportive resources. Five NGOs comprising staff with and without personal experience of PMID, and one family carer, comprised a project advisory group (PAG) who contributed their expertise to the project. They advised on project materials, recruitment, creation of the topic guide, findings and contributed to dissemination activities.

### Ethical considerations

The study received ethical approval from the Faculty of Medicine Health and Life Science’s ethics review board (Ref: MHLS 21_38) at the Queen’s University of Belfast. Participants were provided with an information sheet prior to participation which explained what the study involved, their right to withdraw and the limits of confidentiality. All participants were required to provide written consent prior to participation. This research was conducted in accordance with the Declaration of Helsinki [12].

### Data analysis

The six steps of thematic analysis [13] were used to inductively analyse our data. Three members of the research team independently read, coded the transcripts and met to discuss these. The resulting themes were decided upon collectively, reviewed and agreed. The themes were then defined and the findings were written up for this manuscript. The resulting themes represent important patterns from the data.

### Rigour

We undertook a number of activities to ensure our research was conducted in a rigorous manner. Prior to commencing data collection, the study protocol was reviewed by researchers who were not involved with the research [14]. Further, the protocol and all ethical materials were reviewed by the study PAG and an independent ethics review board at the corresponding author’s institution [15]. To ensure consistency in data collection, the same researcher conducted all the focus groups [16]. Recordings were transcribed verbatim by a transcription service who were required to sign a confidentiality agreement [17]. Three members of the research team took part in the data analysis which increased the credibility of our findings [18]. Lastly, we provide a number of direct quotations from our participants to evidence confirmability [15].

### Findings

Thematic analysis resulted in four primary themes and four subthemes which are summarised in Table 2 and explained below.

**Table 2:** Focus groups themes and sub-themes

Themes	Subthemes
1.0 COVID as a double-edged sword	*1.1 COVID as a catalyst for change*
	*1.2 Challenges during COVID: dealing with change*
	*1.3 Challenges during COVID: fear of COVID*
	*1.4 The online environment: the new normal*
2.0 The struggle for support	*2.1 Invisibility of male carers*
	*2.2 Carers supporting carers*
	*2.3 The only service you get is lip service: non-existent services*
	*2.4 Knowing your rights*
3.0 Constant nature of caring	*3.1 Emotional response to the caring role: Feeling devalued*
	*3.2 Emotional response to the caring role: Desperation of caring*
	*3.3 The multiple demands of the caring role*

Our analysis identified three overarching themes: (1.0) COVID as a double-edged sword, (2.0) the struggle for support, and (3.0) the constant nature of caring (See Table 2). Carers described the COVID-19 pandemic as a double edged-sword, in that it was both at times a catalyst for change whilst also being extremely challenging. Online services became the new normal during the pandemic, again, raising both opportunities and challenges. Carers talked about the ongoing struggles they faced with accessing support. Specifically, the invisibility of male carers, the need for peer support, the lack of service provision for carers, and the importance of carers understanding their rights. Carers also acknowledged the constant nature of their caring role. They described feeling devalued, desperate and struggling to manage the multiple demands of their caring and other important roles. Each of these themes will be explored below.

#### 1.0 COVID-19 as a double-edged sword

Carers talked about COVID-19 being a double-edged sword, in that lockdown and the pandemic had brought about both positive and negative experiences. For some carers COVID-19 was a catalyst for positive change, while for others it was “a complete nightmare” (Female carer, aged 35–44, NI). Carers discussed the challenges in dealing with the changes that lockdown restrictions and the pandemic brought, as well as the fear of themselves or a family member contracting COVID-19. In addition, carers considered how throughout the pandemic, online services and engagements had become ‘the new normal’. Again, for some this was a welcome change that had many positives, for others the online environment brought challenges. These themes will be discussed in turn.

#### 1.1 COVID-19 as a catalyst for change

While the majority of respondents emphasised the challenges discussed in subsequent sections, a significant minority framed the pandemic as a catalyst for positive change in their experiences of family and of care. The COVID-19 lockdown required people to stay at home, leaving schools closed and many furloughed or working remotely. For some carers this resulted in an opportunity for family time which they would not have usually had. Families isolated or ‘bubbled’ together, not seeing the outside world for weeks. For some this meant having older children back at home, partners off from work or working remotely at home; offering a unique opportunity for uninterrupted time, as one carer said:

We are busy every day and then COVID hit, everything stopped and [Daughter] was at home all the time. I was lucky actually because her brother and sister came home so it was nice to have people here, I was lucky because everyone was at home and I really like that (Female, aged 45–54, Scotland).

Not only did the carers talk about the benefits of lockdown for increasing family bonds; they also talked about lockdown providing time for them to develop new skills, a time to get out into nature and try new creative outlets, such as online poetry or art classes. For some, the lockdown forced them to stop, re-evaluate and reflect on their life:

It was a chance for us all to recalibrate, things like your job, things that were important to you and suddenly everything was off the table and there is no one you know, suddenly all things that seemed important actually weren’t as important and so it was a recalibration (Male carer, aged 55–64, Wales).

#### 1.2 Challenges during COVID-19: dealing with change

While, as in other studies, some carers reflected on the positive aspects of lockdown, it was far more common for carers to report heightened and significant challenges associated with COVID-19. For some the pandemic and lockdown was “*a complete nightmare*” (Female carer, aged 35–44, NI). Carers felt trapped in their home, isolated, and dealing with stressful changes to their normal daily life. As above, the pandemic resulted in many families isolating together, with a closure of schools and work. However, these closures included vital services for carers and the people they cared for. These changes in daily routines had significant impact on all. Carers discussed noticing a regression in their family member with ID, including a regression in sleep, diet, behaviour, and speech.

Carers expressed the challenges in their loved ones’ understanding and adaptation to the changes that were happening. Carers talked about services being stopped overnight, schools closing, day centres closing and respite being postponed; a number of sudden changes that all happened simultaneously. The link between these changes and COVID-19 were difficult for the carer to explain and for their family member with ID to understand, having a detrimental impact on all of the family:

He has been sitting at the window every morning waiting for his bus for day service to come and it hadn’t turned up. He had been upset every single morning (Female carer, aged 45–54, Republic of Ireland).

For those carers who had loved ones in residential care or hospital, things were equally difficult. Lockdown, and the changing regulations on visitors to residential homes and hospitals, sometimes resulted in families not seeing their family member for weeks or months. Unsurprisingly this was deeply challenging and at times “*traumatic*” (Female carer, Scotland, aged 45–54), for carers, their loved ones, and the whole family. Carers talked about the impact this had on them and their family member with ID:

His mental health rapidly deteriorated, he became so anxious and depressed because he could not understand why he was separated from his family…… So, if you want my story, it was horrific, absolutely horrific. It caused real damage to my mental health and trauma and huge trauma for my son and others like him in those settings (Female carer, aged 45–54, Wales).

Despite all the changes taking place during the pandemic, carers also talked about the similarities between lockdown restrictions and their daily pre-pandemic lives. Carers talked about having always felt restricted in their daily life given their caring responsibilities. It had never been easy for carers to go out for dinner, go on holiday, and infection control was part of their life even before COVID-19. Thus, what felt like restriction to other people, felt like normal life for carers:

All the talk was that we can’t do this or we can’t do that, we can’t go on holiday and I just thought well we don’t do any of these things anyway, maybe people are getting a bit of a taster for what it is like for other people (Female carer, aged 55–64, England).

#### 1.3 Challenges during COVID-19: fear of COVID-19

In addition to dealing with the many changes due to the pandemic, carers talked about the fear and terror they felt about COVID-19. Carers were frightened of the potential consequences for their family members contracting COVID-19, fearing that they would become severely ill or even die. This was an immense worry for carers, who expressed living in terror during the pandemic:

Then at the beginning of the pandemic my daughter was put on the severe risk list………It was terrifying actually. We ended up isolating, just the 5 of us in our home and our garden for 6 months. We didn’t leave the house…. we lived in fear that anyone we saw, we would get COVID from and [Name] wouldn’t make it through (Female carer, aged 35–44, England).

Carers also feared that they themselves would contract COVID-19, dreading the consequences of being too ill to fulfil their caring responsibilities. For carers, their family member with ID relied on them completely, with few carers having ‘safety nets’ such as respite, or people to fill in for them should something happen. This was a very real worry for carers, and further showcased the vital role they play but also the fragility of this role.

Carers’ fear was further compounded by the mixed messages they felt they received from services. Carers talked about the continual change in visitor policies in hospital or residential homes, and the lack of consistency between services or Health and Social Care Trusts in their policies on COVID-19. Due to this, carers feared their family members needing to attend hospital, and the uncertainty of rules and restrictions on accompanying them:

My biggest fear I suppose was COVID getting into the house…….What would happen to my boys if I got sick or what would happen if one of them got sick and had to go to hospital, I would have to go with them. They wouldn’t cope on their own and the hospital certainly wouldn’t cope with them (Female carer, aged 45–54, Republic of Ireland).

Despite their fear about COVID-19, carers, their family member with ID and their wider families still had ongoing needs. This presented a difficult decision for carers, in that they had to balance the risk of contracting COVID-19 with their own needs and that of their families. For example, some day centres, schools, and respite continued to operate throughout the pandemic, which offered a valuable service for carers and their family member with ID. However, accepting these services potentially exposed their families to a greater risk of contracting COVID-19. Thus, carers were left with a difficult decision to make:

They were frightened about sending their child in. I suppose we were too on one level, but it was the lessor of two evils is the only way I can put it”(Female carer, aged 55–64, England).

#### 1.4 The online environment: the new normal

During the COVID-19 pandemic online engagements became the new normal. For carers, some regarded online services and communication as “*a lifeline*” (Male, aged 55–64, England) to the outside world. Carers talked about engaging in online activities and services such as cooking courses, art classes, poetry, counselling, dancing and much more. Having services and activities online meant that for those carers who found it difficult to get out of the house, even prior to COVID-19, now had the option of engaging in activities or services from their own home. Some carers also talked about online services saving on travel time to and from appointments which also helped reduce costs. In addition, online services and activities opened up an array of opportunities for those families who were shielding, without which, would have had limited or no contact with the outside world. Thus, for some carers the move to online activities and services was deeply appreciated:

Without Zoom I don’t know what would have happened to be honest because there would have been no bingo, cooking, dancing and people making it up as they go along but it really helped…….Zoom sessions were amazing and we wouldn’t have got through it without the Zoom (Male carer, aged 55–64, England).

Not all carers had positive experiences of the online environment. Some carers expressed issues with Information Technology (IT) literacy which made access to online programmes, services and activities more challenging. In addition, carers also talked about how different disabilities can impact a person’s ability to engage online:

learning about Zoom, learning about all of the other issues that come in with that particularly when you are not I.T. literate. That for us was very difficult at the beginning and not only did I find myself trying to teach other people of my age group who had absolutely no idea how to get onto the internet or how to make contact, how to order food stuff and so there was a major gap in the system for people of our age never mind people with children or adults who have severe disabilities (Female carer, aged 65–74, Northern Ireland).

While carers found online engagement beneficial during lockdown, there was concern that despite restrictions being lifted in the UK and Ireland, there was no return to face to face services. Carers discussed the importance of face to face contact and were concerned that given the success of online services during COVID-19, this would now be the ‘new normal’ in a post COVID-19 world:

A lot of our stuff is now only available online, they are not really getting anyone together and I think that although it worked really well during the pandemic but coming out of COVID we need to make sure we are getting back together and supporting each other and really seeing people face to face again (Female carer, aged 35–44, England).

#### 2.0 The struggle for support

#### 2.1 Invisibility of male carers

Our focus groups included 23 male carers. Male carers expressed specific gender needs and experiences of support and services. Male carers discussed their feelings of being overlooked by services due to being male and feeling invisible:

when you go to meetings and you are there and you are as involved, not more but not less and you are literally overlooked sometimes, it’s as though you are physically not there (Male carer, aged 45–54, Wales).

Male carers expressed having different needs than female carers, however there were little to no services available to meet this need. Services were felt to be predominately orientated towards female carers, with the role of male carers being seen in some way as second class to that of a female. Male carers wanted recognition that they have the same role and responsibilities, face the same stressors and challenges as female carers, and should not be seen as fulfilling a lesser role solely based on their gender. In addition, male carers wanted more opportunities to meet and gain support from other male carers. Peer support was something that was more widely available for women, however, this was seen as vital to supporting men:

There is dads involved in that and that’s what we are looking at is to open up a parent café that is specifically for dads because they do respond differently and they do have different needs to mums as well (Female carer, aged 45–54 Wales).

#### 2.2 Carers supporting carers

The value in peer to peer support was not a unique theme to male carers. All carers spoke of the vital nature of carers supporting other carers. Carers talked about the comfort of knowing other carers who shared their experiences, their stressors, their hopes and worries. There was comfort in the fact they did not have to explain themselves, due to an unspoken understanding between carers:

Whereas when people are in the same position as you and that’s probably one of your biggest strengths is to have people who are in similar minded situations and to feed off their advice and support level and understanding because then you are not trying to explain to all these people about your son or daughter’s needs because they know, they are there (Female carer, aged 35–44, Republic of Ireland).

Carers not only had a deep understanding of other carers’ situations, they also provided a source of crucial information. Carers discussed learning key information and receiving support from carer support groups, online forums, or just informal conversations with other carers. Carers received vital information on an array of topic such as benefits, entitlements, respite, transitions, diet, behaviour management, and much more. Carers described this support and information as vital because they felt they would not have been able to get this from any other source:

I didn’t know what I was entitled to and there was no support from professionals, social services or the medical side to be honest and so really I found all the useful day to day stuff from other parents through joining parent support groups (Female carer, aged 55–64, Wales).

It was clear from our focus groups that carers wanted more opportunities for peer support. Some carers had been in their caring role for 20 plus years and had developed an expert wisdom through their experience that they wanted to pass on and share with carers who were at earlier stages of their journey. Carers appreciated the learning they received from other carers and felt they wanted to pass this on to others:

it was another parent that gave us that crucial bit of information that helped so perhaps we can do the same for one another (Female carer, aged 35–44, England).

#### 2.3 The only service you get is lip service: non-existent services

One of the largest themes identified from our focus groups was the frustration and anger at statutory services. Carers talked about the continuing and ongoing fight for services such as respite, benefits, day centres, counselling, occupational therapy, physiotherapy and more. These services were viewed as essential, however, frustratingly families were not automatically entitled to receive them. When carers requested support, they felt the default answer from services was always no. In many cases this resulted in a fight to prove entitlement:

The amount of additional stress it causes when the default is always no. You go to ask for something and you know you really need it because you are not going to survive without it and you know that the first answer you get is no(Female carer, aged 45–54, Republic of Ireland).

This continuing and often long term fight for services, was exhausting and extremely stressful, creating feelings of anger and frustration at statutory services. Carers expressed a sense of exceptional stress, as a result of this ongoing fight for vital services: “my hair is falling out from the stress” (Female carer, aged 55–64, Republic of Ireland).

Carers discussed specific difficulties with access to adult services. Specifically, they felt a dramatic shift in services and support following their family members’ transition from children to adult services; with adult services having a dearth of available supports. Carers discussed the sudden nature of this transition:

that’s what happens once they transition to adult services. I think the system expects your child to miraculously recover and they haven’t got any disabilities anymore once they reach 18 and there is nothing really out there, no support for when they turn past 18 (Female carer, aged 45–54, England).

The COVID-19 pandemic further compounded carers’ frustration with services. Carers described services withdrawing at the beginning of the COVID-19 pandemic due to restrictions. However, they felt that COVID-19 was now being used as an excuse for services not returning to normal, pre-pandemic levels, despite restrictions being lifted. This delay in the reinstalling of services left carers with an extra burden of care, that they felt was unmanageable:

Those services are nowhere near returning back to pre-pandemic and what they were before. To be perfectly honest, I am a parent, I’m not an occupational therapist nor am I a physio. There has been no professional oversight from any of these services for a prolonged period of time and personally I think it is awful. All it does for me is just gives me something else to worry about at night in a nutshell (Female carer, aged 45–54, Scotland).

#### 2.4 Knowing your rights

With a lack of support, carers felt that if they needed services they would have to fight for these themselves. Carers discussed the responsibility being solely on their shoulders which was a very isolating experience:

There wasn’t really a lot there, it kind of felt like it was me having to do everything and the responsibility was totally on me and it just really makes you feel so alone that if there is anything like that it is just you. The help is not there so it does make you feel a bit doom and gloom to be honest (Female carer, aged 45–54, England).

Carers agreed, if they had to fight for services themselves then it was essential that they were well informed about their rights and entitlements. Knowing your rights and what carers were entitled to, was thought to alleviate feelings of being alone:

just knowing what your rights are so you don’t feel alone and you are sort of educated in what you are entitled to (Female carer, aged 45–54, England).

Part of addressing the need for carers to know their rights and what they were entitled to, was having ease of access to this information. Carers had little spare time, so important information needs to be accessible and easy to interpret. In addition, carers talked about not having time to go and look for lots of different types of information on entitlements and legal rights. Carers discussed the usefulness of having important information all in one place:

It’s just bringing all the information together on one kind of website or something would be really helpful for carers because they are dotted everywhere and you don’t really know where to get help or information from (Female carer, aged 45–54, England).

#### 3.0 Constant nature of caring

#### 3.1 Emotional response to the caring role: feeling devalued

Carers discussed their emotional experiences of their caring role. Carers talked about the lack of understanding and value given to the caring role, feelings of powerlessness, not having a voice, and a loss of identify.

Carers expressed feelings of not feeling heard either by services or government. As discussed above carers had to fight for support, however, they often felt that their pleas were ignored, leaving them feeling powerless. Carers discussed being left out of decision making, with their views and wishes falling on deaf ears:

Our voice isn’t being heard and that’s not acceptable that you can just move things left, right and centre and I’m sorry you are a carer, it’s your job and it’s your family member, it’s up to you and you are left with nothing (Female carer, aged 65–74, Northern Ireland).

In addition, carers did not feel that their role was valued by services and more widely, society. This resulted in some carers feeling worthless, despite recognising the vital nature of the caring role. Feeling de-valued by society, led to carers feeling angry and upset:

I just feel worthless, valueless and I shouldn’t feel like that because I am saving them a fortune and my life is on hold. I know she is my responsibility and I get that but no, these past 2 years I really have struggled and I just feel very cross, angry, upset, every negative word that you can think of (Female carer, aged 55–64, Northern Ireland).

The caring role was seen as all consuming, demanding and lifelong. For carers, this was just part of their normal life. The carers were so accustomed to putting their family before themselves, with their caring role taking priority above and beyond all other aspects of their lives. For some carers, their caring role had become the entirety of their identity:

As the years go on you just realise that you have slowly lost your identity. What do I like to do? Well that’s not really relevant and it’s what I need to do for my children (Female carer, aged 45–54, Scotland).

#### 3.2 Emotional response to the caring role: desperation of caring

The COVID-19 pandemic pushed carers to their limits, with many feeling exhausted and burnt out. A contributing factor to this exhaustion, was the overwhelming sense of responsibility carers felt over the COVID-19 pandemic with little to no support. Carers had been pushed to the edge of how much stress they could tolerate due to the COVID-19 pandemic, impacting both the carers’ mental and physical health.

The COVID-19 pandemic was difficult for everyone, but specifically for family carers. Due to restrictions, isolation and withdrawal of services, carers were pushed to the limits of what they could cope with. Coming out of the COVID-19 pandemic carers expressed feeling exhausted, burnt out and described being in a state of desperation:

I’ve never been so tired in my life and I have been doing this for 16 years, but I have never been as tired as I am now and it’s just so hard with no end in sight (Female, aged 55–64, Scotland).

One of many contributing factors to this exhaustion was the overwhelming sense of responsibility carers felt during lockdown. COVID-19 restrictions meant that services stopped, day centres closed, schools closed, and families isolated at home. This placed further burden on carers to step in to address the shortfall, increasing their sense of responsibility, which some found overwhelming:

I think I would say for the first months throughout the first lockdown I must have went through every stress and emotion. I’m a pretty calm person normally but I was really quite stressed because I have total sense of responsibility for two young people (Female carer, aged 65–74, Scotland).

The consequences of this, was that carers’ mental and physical health suffered:

For me and my mental health it has a knock on impact on everybody else in the household, I would say the input of [Local charity] kept me alive in terms of stress, anxiety and everything else that was going on (Female carer, aged 35–44, Northern Ireland).

#### 3.3 Multiple demands this research employed focus gof the caring role

The caring role was perceived as a challenging one. However, caring responsibilities were often just one more thing they had to juggle among an array of competing demands. The carers who took part in this research cared for people with profound and multiple disabilities, which meant that the care they provided for their family member was often complex and required they develop specialised skills:

If you are a carer to somebody with profound learning disabilities who has all these healthcare needs as well, that’s a 24/7 job where you don’t get much let up at all. There is no burn out option for families (Female carer, aged 55–64, Scotland).

However, carers often cared for more than one person, and had to juggle these caring responsibilities with other demands such as, their own health issues or employment. In addition, carers had the responsibility of worrying about their whole family:

I worry about all of our health, I worry about my health, I worry about my ability to stay alive as much as possible (Female carer, aged 45–54, Scotland).

As discussed previously, the caring role is a lifelong commitment meaning that carers often worried about what the future would mean for their family members with ID. Carers were often parents caring for their children, so inevitably future care plan was an ongoing concern. In addition to future care planning, carers also had to consider contingency plans in the event they were to become ill. Carers felt that being ill was a luxury that they could not afford due to the demands placed upon them:

You haven’t got time to be sick, you haven’t got time to look after yourself when you are putting your loved one first. It’s not like you say a conscious thought, it’s just the way it is and then it’s like you say until your body has literally shut down and can’t do anymore, it’s screaming at you to say I am physically exhausted. If you are mentally exhausted you are physically exhausted, how you are supposed to do self-care when you haven’t got the time because you are too busy caring for somebody else (Female carer, aged 55–64, Scotland).

## Discussion

This study sought to explore the experiences of family carers of those with PMID during the COVID-19 pandemic. These individuals represent an underserved and under researched group who provide vital care and support for their family members. Previous research had explored the impact of the COVID-19 pandemic on family carers, through online surveys [6, 7, 19, 20] or small scale qualitative studies [2, 10]. Our study adds to this evidence through adopting a large scale qualitative approach, adding further depth of understanding of the experiences of family carers’ of people with PMID. There is consensus within the literature that the COVID-19 pandemic had an adverse impact on family carers’ mental health and burden of caring responsibilities [6, 7, 19], which our study can corroborate. Our study adds to these findings, highlighting that carers and their families struggled with adapting to the changes in routine and the intense fear of contracting COVID-19. However, whilst the COVID-19 pandemic was undoubtedly a traumatic time for many carers and their families, for some it was also a time for family connection and reflection. For some, it was an extension and intensification of negative experiences that were there before the pandemic. To understand the experiences of an event it is important to have a sense of what life was like before it. Many carers felt that their lives had always been difficult for example in looking for the right support or in having a life beyond caring. The pandemic seemed to remove some of the pressures of daily life that family carers had experienced prior to the pandemic. There was time to devote to personal interests (albeit online) and the needs of family members could be attended to without extraneous demands. There is no question that the impact of the COVID-19 pandemic is still being felt by family carers.

Our findings showed that the COVID-19 pandemic offered a unique opportunity for online services to become the new normal for carers. This offered both new possibilities and challenges. Previous research also recognises that online support and services became the standard medium of care during the pandemic [21], offering opportunity to develop new and innovative ways of supporting family carers [20, 22]. These new opportunities, opened up possibilities for carers to meet virtually, develop friendships and a sense of community [20, 22]. Online support was not only useful, for some it was a lifeline to the outside world throughout lockdown periods. However, remote services were not viewed as beneficial by all carers. Previous research has identified that family carers experiencing financial difficulties experience more problems with accessing online services [20]. The current study found that in addition to financial barriers, information technology literacy and disability of some family carers were additional barriers to accessing online services and support.

Despite promising opportunities for statutory services to be delivered remotely, family carers continued to experience difficulties in accessing them. Our findings highlight that carers experience an ongoing fight for service provision, which was often exhausting and distressing. Carers’ frustrations with service provision is well documented within the literature [23, 24], with the pandemic further increasing these issues [7, 20]. Our findings indicate that male carers in particular, struggled to be acknowledged by services, feeling they are not recognised nor heard. To the best of our knowledge, this is one of the first studies to specifically consider the experiences of male carers in the context of people with PMID. Previous research has explored the experiences of male carers in other contexts, such as, Autism [25], Multiple sclerosis [26], and dementia [27]. Previous research indicates that male carers may have different needs to those of female carers, find it harder to ask for help [25], can feel more isolated than their female counterpart, and are more likely to continue to work full time in addition to caring [26]. Male carers wanted services and supports that were specifically tailored towards men. Furthermore, they wanted opportunities to meet other male carers, to share experiences and offer peer support. We recommend that further research is needed to understand how services can meet the specific needs of male carers.

All carers acknowledged the vitality of the support carers could offer each other; gaining value from shared experiences. A review examining peer support interventions for carers of children with complex needs found that carers perceived peer support interventions as valuable but found no evidence on the effectiveness of these interventions [28]. The lack of effectiveness studies is not surprising given the often informal nature of this type of support. Despite this, there is qualitative research on carers’ experiences of peer support in the context of dementia [29–31]. This research identified that peer support can be an important source of emotional and social support [29], can improve self-efficacy [30], and promote resilience and social interactions [31]. Similarly, our findings indicate that peer support is perceived by carers as highly valuable, in relation to learning from others and developing a sense of belonging and community. Carers want more opportunities to engage with each other, face to face or online.

### Policy implications

The findings of this study echo parallel studies of the difficulties faced by carers during COVID-19 and related lockdowns, and suggest the strong potential for a significant negative long-term impact of the pandemic on the mental health and wellbeing of carers who supported family members with profound and extensive needs. It is clear therefore that policy and services must retain focus on family carers and their needs, and include the needs of family carers within all future health and social care initiatives. This is necessary due to the impact of the pandemic on their health and well-being and ability to continue to provide the level of care and support required by their family member with PMID [32]. Failure to recognise and address the care and support needs of people with PMID and their families within policy initiatives and developments will add to their burden of care and place further potentially avoidable impact on health and social care services. Ensuring there are interventions and supports that are reflective of their specific needs, whilst recognising that family carers are themselves diverse and possess diverse needs, are therefore required as part of policy initiatives.

It is also clear that online services played a useful role during this period, and it is fair to assume that many of the benefits of such support can be sustained and built upon during this period. Reflections on the experiences of family carers can ensure that online supports, necessarily developed at pace during the pandemic, can now be enhanced and improved, so as to offer both routine and ongoing day-to-day support, as well as enhanced provision should we be faced with a comparable national emergency. Those family carers who engaged well with online platforms saw the value of these in connecting them with NGOs and in accessing supportive resources. Of particular pertinence may be the use of digital communication to bring otherwise isolated carers together to share experiences and provide mutual support, especially for male carers.

It is equally clear, however, that we cannot now default to online provision as the primary means of support to family carers of those with PMID. While it may prove an effective support for many, it remains inaccessible to others. The experiences of our participants were such that the normalisation of online support is infeasible without systematically addressing barriers relating to poverty and financial disadvantage, access to and literacy in digital and information technology. Particular disparities are apparent in the availability of cost effective and reliable internet provision.

Policy makers and service commissioners should therefore consider the lifeline that internet services can provide to family carers who may come from lower socio-economic backgrounds, live in rural communities or have poor digital literacy. By providing affordable services and targeted training, policy makers could improve how marginalised family carers engage with online resources to support their families. Further research on the experiences of such marginalised individuals would provide the necessary evidence to assist policy makers to provide funding and training to address this need.

### Strengths and limitations

This manuscript presents important and unique information on the lived experiences of family carers of people with PMID from across the four countries of the UK and the RoI. As such we have gained a broader perspective of life during the COVID-19 pandemic and also beyond it. Our use of focus groups allowed for a greater depth of explanation in our data when compared to many of the surveys conducted on this topic. While other qualitative studies have emerged following the COVID-19 pandemic these have tended to be smaller in scale and focused on intellectual disability rather than PMID. A further strength of this work relates to the large number of male carers who provided their unique perspectives on the caregiving experience. The voices of male carers are often absent from the literature and this research has been greatly strengthened by their involvement. Our project also captured a broad age range of participants which increases the likelihood that our sample is representative of family carers. However, as only around 22% of participants came from black and minority ethnic backgrounds we cannot claim that our sample is representative of family carers in the UK. Black and minority ethnic carers may not be well represented among those receiving support through NGOs included in this project or may have chosen not to participate in this research. A further possible limitation of this work relates to our limited success with recruitment from the RoI. It would have been preferable to increase the numbers of family carers from the RoI to ensure the experiences captured here were representative of this country. However, data were analysed collectively and we are confident that the perspectives of family carers from the RoI did not diverge from those of carers from the UK. All our participants were recruited through NGOs, meaning each had made a decision to access support. Family carers from black and minority ethic communities, the RoI, those who feel isolated or have limited or no internet connection were less likely to engage with NGOs, and this research. Future efforts to specifically recruit from these groups by connecting with community leaders, advertising research offline and conducting data collection in-person may increase representation.

## Conclusions

Family carers play a crucial role in providing care to their family members with PMID. The caring role is constant and demanding but is also immensely rewarding. The COVID-19 pandemic exacerbated the demands of this role for family carers who felt they were already struggling to receive the services they needed. In a post pandemic world family carers should be provided with improved and tailored services to better support their quality of life. Male cares are an under recognised group who feel excluded from some services and may require bespoke forums through which they can gain support. Further research is required to explore the experiences of male carers to determine how they can best be supported. Female carers also require support and should not have to constantly fight and struggle to receive it.

We do not yet know the long term impact of the COVID-19 pandemic on family carers. The pandemic created innumerable challenges for this population whilst also providing opportunities. It is important that we learn from the experiences of family carers to maximise these opportunities rather than returning to sub-optimal pre-pandemic supports and services.

## Data Availability

Anonymised data are available through the UK Data Archive https://reshare.ukdataservice.ac.uk/856210/ or by contacting the corresponding author.

## Abbreviations

COVID: 19-Coronavirus disease
ID: Intellectual Disability
NGO: Non-government organization
PAG: Project Advisory Group
PMID: Profound and Multiple Intellectual Disabilities
RoI: Republic of Ireland
UK: United Kingdom
